# Insights into biomethane production and microbial community succession during semi-continuous anaerobic digestion of waste cooking oil under different organic loading rates

**DOI:** 10.1186/s13568-018-0623-2

**Published:** 2018-06-01

**Authors:** Jing He, Xing Wang, Xiao-bo Yin, Qiang Li, Xia Li, Yun-fei Zhang, Yu Deng

**Affiliations:** Key Laboratory of Rural Renewable Energy Development and Application of the Ministry of Agriculture, Biogas Institute of Ministry of Agriculture, No. 13, Section 4, Renmin South Road, Chengdu, 610041 People’s Republic of China

**Keywords:** Microbial community, Anaerobic digestion, Waste cooking oil, Biomethane, Organic loading rate

## Abstract

High content of lipids in food waste could restrict digestion rate and give rise to the accumulation of long chain fatty acids in anaerobic digester. In the present study, using waste cooking oil skimmed from food waste as the sole carbon source, the effect of organic loading rate (OLR) on the methane production and microbial community dynamics were well investigated. Results showed that stable biomethane production was obtained at an organic loading rate of 0.5–1.5 g VS L^−1^ days^−1^. The specific biogas/methane yield values at OLR of 1.0 were 1.44 ± 0.15 and 0.98 ± 0.11 L g VS^−1^, respectively. The amplicon pyrosequencing revealed the distinct microbial succession in waste cooking oil AD reactors. Acetoclastic methanogens belonging to the genus *Methanosaeta* were the most dominant archaea, while the genera *Syntrophomona*, *Anaerovibrio* and *Synergistaceae* were the most common bacteria during AD process. Furthermore, redundancy analysis indicated that OLR showed more significant effect on the bacterial communities than that of archaeal communities. Additionally, whether the OLR of lipids increased had slight influence on the acetate fermentation pathway.

## Introduction

It was estimated that annual amount of food waste (FW) come up to 36.4 and 89 million tons in USA and E.U., respectively (Agency et al. 2012; Lin et al. 2013). Approximate 60 million tons annually in China (Meng et al. 2014), including 6 million tons of waste cooking oil. Among the various proposed methods to alleviate these problems, anaerobic digestion (AD) has been considered as the waste-to-energy technology and has been performed well for treating food waste and waste cooking oil (Dasgupta and Mondal 2012; Kim et al. 2008).

Food waste contain three fractions including carbohydrates, proteins and oils with the different biodegradability order of carbohydrates > proteins > lipids (Christ et al. 2000), which means that carbohydrates and protein can be degraded and fermented faster than that of lipids. Hence, lipids degradation is the crucial step in the process of FW anaerobic digestion. High content of lipids in FW could not only impeded digestion rate of FW, but also result in the accumulation of residue in anaerobic digester (Sun et al. 2014; Zhang et al. 2013). Therefore, lipids degradation is critical for effective conversion of FW to biogas.

Lipids have been considered to be a good feedstock for the production of renewable energy at an industrial level. Furthermore, these lipids possess high methane production potential, a factor that may be harnessed for production of alternative fuels through anaerobic digestion. Studies have revealed that AD reactors can convert around 63–98% of fats, oils and grease (FOG) into biogas (Davidsson et al. 2008; Luostarinen et al. 2009). During AD process, the lipids are hydrolyzed to produce glycerol and long-chain fatty acids (LCFA) (Hanaki et al. 1981), which were further degraded through the *β*-oxidation pathway (Angelidaki and Ahring 1992; Cirne et al. 2007). The bacterial species responsible for the syntrophic *β*-oxidation of LCFA in AD reactors belong to two families, *Syntrophomonadaceae* and *Syntrophaceae* (McInerney 1992; Jackson et al. 1999; Hatamoto et al. 2007; Sousa et al. 2007a, b; Wu et al. 2007).

With previous studies, it is proved that the elevated FOG (fat, oil and grease) loading rates would lead to the fails of anaerobic digestion process, and hinder the sufficient methane production, especially co-digestion of FOG with municipal wastewater sludge (Luostarinen et al. 2009; Wan et al. 2011; Girault et al. 2012; Noutsopoulos et al. 2013; Wang et al. 2013). High concentrations of LCFA also suppress the lipid hydrolysis by anaerobic micro-organisms (Lalman and Bagley 2000; Rinzema et al. 1994) and further limiting their bioconversion into methane. LCFA specifically inhibits the activity of aceticlastic methanogens, hydrogenotrophic methanogens and syntrophic bacteria (Pereira et al. 2005).

FOG loading threshold values that cause a reduction in methane yields, especially during co-digestion with wastewater sludge, range from 0.5 to 2.0 g VS L^−1^ days^−1^ (Silvestre et al. 2011). Some of previous studies have proved the biomass adaptation was important for stable FOG digestion with municipal wastewater sludge (Silva et al. 2014). The dynamic of microbial communities and the relationship between the microbial community structure and LCFA conversion efficiency have been previously studied in reactors with co-digestion of FOG with other sources (Ziels et al. 2016).

As is known FOG is neither easily treated by conventional method, nor decomposed biologically, which can be attributed to their inherent tendency and capability to form insoluble aggregates and float on the surface of water (Stoll and Gupta 1997). The stability of FOG AD has become the barriers in practical application. The effect of organic loading rate (OLR) on the microbial community dynamics using waste cooking oil skimmed from food waste as the only AD carbon source has received little attention. To better understand the loading threshold values of FOG, microbial mechanisms research of unstable fermentation during anaerobic digestion of waste cooking oil is imperative.

In this paper, we start an AD reactor with 100-day hydraulic retention time (HRT) at low concentrations of FOG (0.5 g VS L^−1^ days^−1^) for 50 days to acclimatize the inoculum. After the start stage, AD reactor run at HRT of 10-day with the OLR heightened in a step-by-step mode. For each loading segment, the methane production is described through testing the methane yield and VFA concentration. The aim of this study was to elucidate the effect of OLR on the biomethane production and microbial community structure during different phase, and propose the most suitable FOG loadings for waste cooking oil-based anaerobic digestion.

## Materials and methods

### Substrates and inoculum

Waste food (WF) was collected from a restaurant located in ChengDu, China. Waste cooking oil (hereby referred to as FOG) was separated using an oil remover, then precipitated FOG for 48 h to make particulate precipitation, after that filtered to remove bubbles and solid and then stored at 4 °C. The pretreated FOG with density of 0.928 g mL^−1^ were used as the raw material for anaerobic fermentation. The seed sludge was obtained from an anaerobic digester that was used for treating WF at 35 °C. Before being loaded into reactors, the sludge was filtered through a 2-mm stainless steel sieve. The volatile solids (VS) concentration of sludge was 20 ± 1 g VS L^−1^.

### Reactor operations

A semi-continuous complete anaerobic digester (3 L working volume) functioning at 35 ± 1 °C was used in this study. The digester was set to mix the organic waste with its axial flow impellers at 250–300 rpm. The initially loaded anaerobic sludge in the reactor was diluted with tap water to reach 5.4 ± 0.2 g VS L^−1^. The reactor was fed once a day with 100-day HRT at the start stage, then the loading rate was increased step-by-step and the hydraulic retention time (HRT) remain 10-day throughout the experiment, as shown in Table 1.

**Table 1:** The influent FOG, VSLR and HRT in the feed versus time for the digester

Days	FOG VSLR (g VS L^−1^ day^−1^)	HRT (day)	FOG daily added (g)	Water daily added (mL)
0–50	0.5 (0.05)	100	1.5	28.5
51–61	0.5 (0.05)	10	1.5	298.5
62–77	1.0 (0.1)	10	3.0	297
78–91	1.5 (0.1)	10	4.5	295.5
92–103	2.0 (0.1)	10	6.0	294
104–113	0	10	0	300

Values in parentheses indicate one standard deviation

Digester performance was monitored by calculating the daily biogas production, methane content, effluent volatile fatty acids (VFA), total solids (TS) and volatile solid content (VS). The biogas produced was determined with a wet gas meter and collected in foiled aluminum bags (Delin, China). TS and VS contents of the sludge were determined according to the standard methods (AWWA et al. 1995).

The biogas composition (methane, carbon dioxide, and nitrogen) was analyzed daily using a gas chromatography (GC-2010, Shimadzu, Japan) equipped with a thermal conductivity detector, which used hydrogen as the carrier gas. VFA fractions such as acetate, propionate and butyrate were analyzed by chromatography (7820A, Agilent, Palo Alto, CA, USA). The analysis was done using a capillary column (DB-FFAP, Agilent, Palo Alto, CA, USA) equipped with a flame ionization detector.

### DNA extraction and polymerase chain reaction (PCR)

Digester biomass samples were collected for DNA analysis on the 51st, 61st, 68th, 78th, 82nd, 92nd, 98th, 104th and 113th day. 20 mL of digester sludge were transferred into sterilized 50-mL tubes. Then samples were centrifuged immediately at 10,000×*g* for 20 min at 4 °C. The supernatant was decanted and was immediately frozen at − 80 °C. Total DNA was extracted from each sample using E.Z.N.A.^®^ soil DNA Kit (Omega Bio-tech, Norcross, GA, USA). The V3–V4 hypervariable regions of the 16S rRNA genes of the extracted DNA samples were amplified in a PCR system (GeneAmp 9700, ABI, Foster City, CA, USA) with primers 338F and 806R for bacteria and 344F and 915R for archaea.

The PCR reactions were conducted using the following steps: 3 min of denaturation at 95 °C; 27 cycles of 30 s at 95 °C, 30 s for annealing at 55 °C, and 45 s for elongation at 72 °C, and a final extension at 72 °C for 10 min. The extracted DNA was further purified using the Kit (Axygen Biosciences, Union City, CA, USA), as per the manufacturer’s protocols.

### High throughput amplicon sequencing of 16S rDNA genes and analysis

The PCR products were sequenced with Illumina MisSeq platform in Majorbio Bio-Pharm Technology Co. Ltd., (Shanghai, China) A total of 383,117 quality-filtered reads were obtained from the Illumina sequencing of bacterial 16S rDNA gene amplicons (8 samples), while a total of 355,640 quality-filtered reads were obtained from sequencing of archaeal 16S rDNA gene amplicons (8 samples). The raw data were deposited into the NCBI Sequence Read Archive (SRA) database (BioProjectPRJNA386213).

### Statistical analysis

Each experiment was repeated three times using duplicate samples. Statistical comparisons were made by the analysis of variance (ANOVA). Differences were considered significant when the p-values were < 0.05.

## Results

### Bioreactor performance

**Fig. 1:**
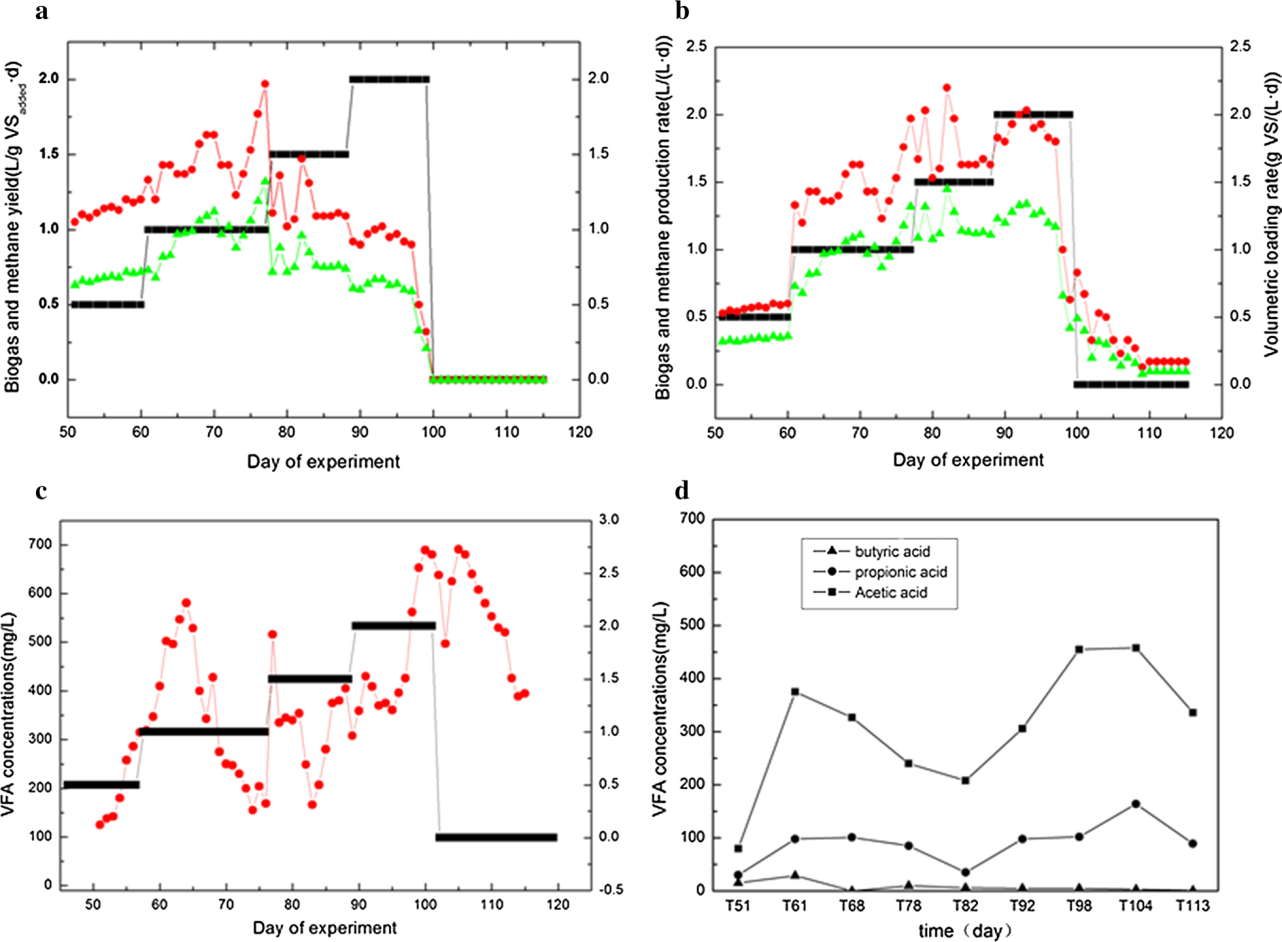
Evolution of biogas/methane yield (**a**), volumetric biogas/methane production rate (**b**) and volatile fatty acids (VFAs) (**c**, **d**) in reactors with elevated OLR

Semi-continuous anaerobic digestion of FOG started at low OLR (0.5 g VS L^−1^ days^−1^) for 50 days. SBY/SMY profiles of the added FOG, VS/volumetric biogas/methane production rate, VFAs and methane concentration from 51st to 114th day were shown in Fig. 1. Performance oscillations in terms of SBY/SMY and VFAs concentrations were observed in the reactor at each elevation of OLR, thus implying internal adaptation of microbial communities.

At the end of the acclimation phase, elevation of OLR resulted in a slight increase in the average SMY or performance at OLR of 1.5 g VS L^−1^ days^−1^. However, the performance was found to decrease when the OLR was added up to 2.0 g VS L^−1^ days^−1^. The calculated values of average SBY and SMY at OLR 1.0 g VS L^−1^ days^−1^ were 1.44 ± 0.15, and 0.98 ± 0.11 L g VS^−1^ days^−1^ (average of the data obtained from 61st to 77th day). Subsequently, at OLR 1.5 g VS L^−1^ days^−1^, the average SBY and SMY were found to be 1.64 ± 0.11 and 0.78 ± 0.06 L g VS^−1^ days^−1^ (average of the data obtained from 78th to 88th day), whereas the average SBY and SMY values obtained at OLR of 2.0 g VS L^−1^ days^−1^ were 0.85 ± 0.16, and 0.56 ± 0.10 L g VS^−1^ days^−1^ (average of the data obtained from day 89th to 99th).

The VFA concentration profiles indicated that the reactor maintained a fluctuating state during OLR elevation, as shown in Fig. 1c. Furthermore, at OLR 1.0 g VS L^−1^ days^−1^, VFA concentration was found to be less than 300 mg L^−1^, which indicated good adaptation to the first elevation in OLR. The VFA concentration further rose to 600 mg L^−1^ as the OLR was increased to 1.5 g VS L^−1^ days^−1^, after which it quickly fell to 150 mg L^−1^. Interestingly, the VFA concentration again increased to 400 mg L^−1^, which indicated that accumulation of VFA in the reactor showed no influence on the fermentation balance. Once the OLR increased to 2.0 g VS L^−1^ days^−1^, VFA concentration was limited to 400 mg L^−1^, and then rapidly increased to 700 mg L^−1^, thereby bringing down the biogas production in an abrupt manner. VFA concentration was gradually reduced to 400 mg L^−1^ by the 113th day when no organic waste materials were further fed.

### Bacterial community dynamics as revealed by high-throughput pyrosequencing

High-throughput pyrosequencing is one of the most widely used methods of investigation of microbial community structure and dynamics. In the present study, the samples collected on day 51st, 61st, 68th, 78th, 82nd, 92nd, 98th, 104th and 113th (which were denoted as T51, T61, T68, T78, T82, T92, T98, T104 and T113, respectively) were used for analysis. The results obtained from the analysis of various diversity estimators viz. Chao1, Shannon index, ACE, Simpson index and Good’s coverage of sequencing for each sample are shown in Table 2. The coverage of sequencing for the all the samples were found to be more than 0.99, which integrated with higher ACE, Chao1 and Shannon index values. These values jointly implied the presence of highly diverse bacterial communities in T51, T61, T68, T78, T82 and T113. The high diversity of microbial communities can be regarded as an augment for functional redundancy, at least in terms of ecology (Finlay et al. 1997). These values also showed the low diverse bacterial communities at T92 and T98 caused by load shock, that presumably means the change of microbial communities abundance and reduction of stability.

**Table 2:** Statistics analysis of the bacterial 16S rRNA gene libraries obtained from the pyrosequencing

Sample ID	ACE	Chao1	Coverage	Shannon	Simpson	Sequences
T51	306	311	0.99	3.16	0.09	36739
T61	330	334	0.99	3.92	0.04	34919
T68	329	330	0.99	3.76	0.04	28493
T78	317	322	0.99	3.60	0.06	41490
T82	323	318	0.99	3.47	0.05	28646
T92	272	392	0.99	2.39	0.21	39491
T98	259	259	0.99	2.82	0.14	29366
T104	270	260	0.99	2.13	0.31	36147
T113	304	310	0.99	3.03	0.09	42475

All values were calculated at 0.03 distance limit

**Fig. 2:**
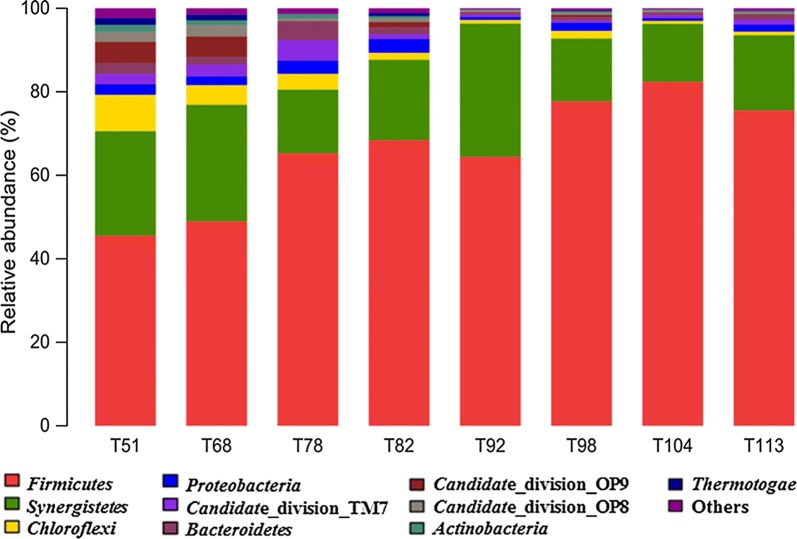
Taxonomic compositions of bacterial communities at phylum level in each sample retrieved from pyrosequencing. The number in the sample names represented the day when sampling occurred

Distribution of sequences at the phylum level in each sample was shown in Fig. 2. The sequencing results indicated that there were 10 phyla in total, each with a relative abundance higher than 1% in at least one sample. The minor phyla were grouped into a separate group. *Firmicutes* was the most dominant phylum that appeared in the entire process, while *Synergistetes* was the secondary. *Chloroflexi* and *Bacteroidetes* possibly playing a necessary role in the community dynamics appeared throughout the process with lower percentages.

**Fig. 3:**
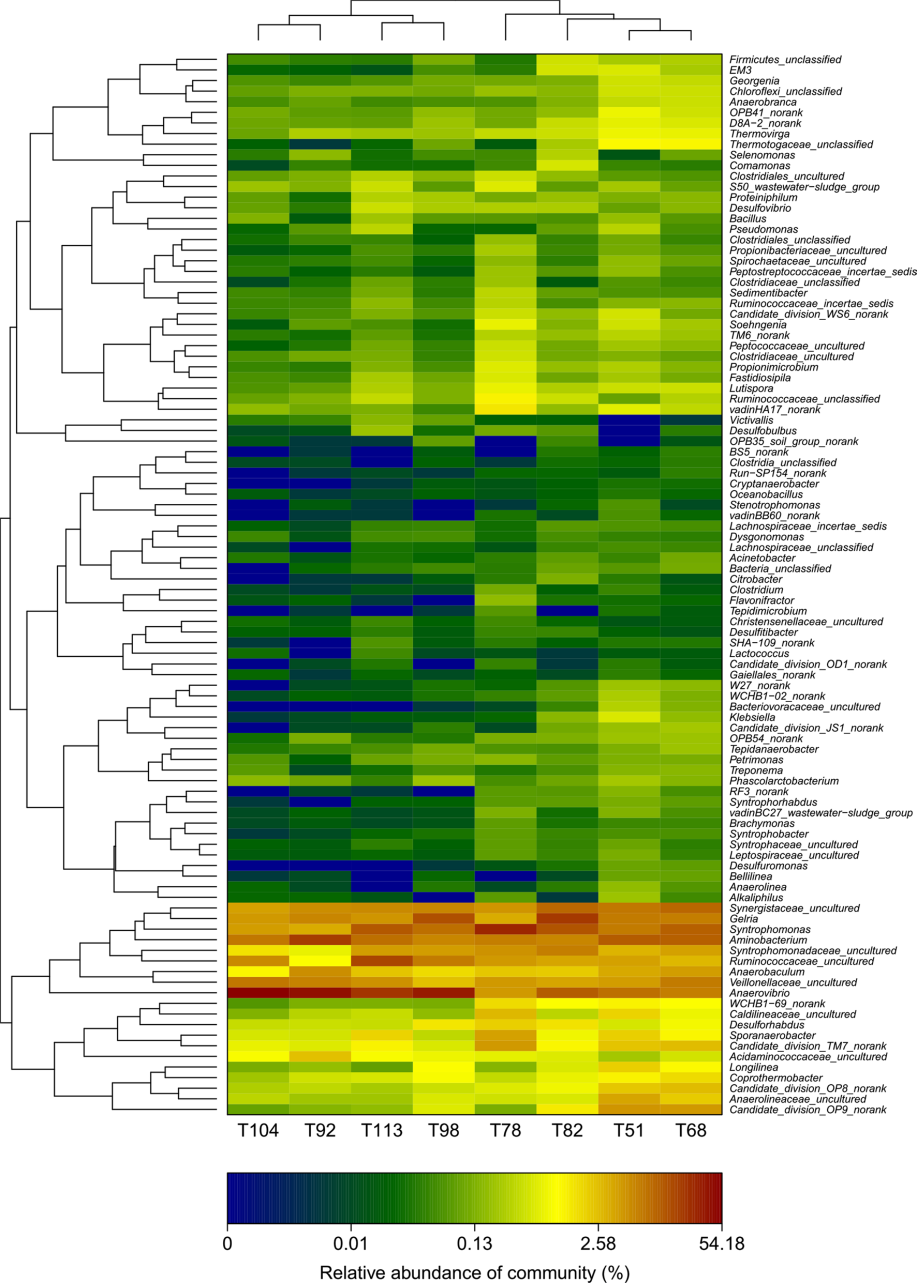
Hierarchical cluster analysis of microbial communities among the 8 samples. The Y-axis is the clustering of the top 100 abundant genus. Different samples were clustered based on complete linkage method

To further integrate the dynamics of bacterial communities, the sequencing data was deconstructed at the subdivision level. Therefore, the relative abundance of each genus in all the samples was calculated. The top 100 genera were detected, among which 147 genera with a proportion higher than 0.5% was screened as the abundant genera (Fig. 3). Other genera were considered as minor groups. The sequence distribution at the genus level in each sample are shown in Fig. 3. *Anaerovibrio*, *Syntrophomonas*, *Aminobacterium*, *Gelria*, *Synergistaceae*, *Longilinea* and *Ruminococcaceae* were the major genera that appeared during the entire process. *Anaerovibrio* emerged as the most dominant genus with increasing organic loading rate (82nd to 113th day) and possessed high tolerance throughout the process. On the other hand, the proportions of *Anaerovibrio* and *Aminobacterium* increased initially and then decreased with elevation of OLR rates. The proportion of *Longilinea* decreased, while *Ruminococcaceae* increased with time.

### Dynamics of methanogen communities

**Fig. 4:**
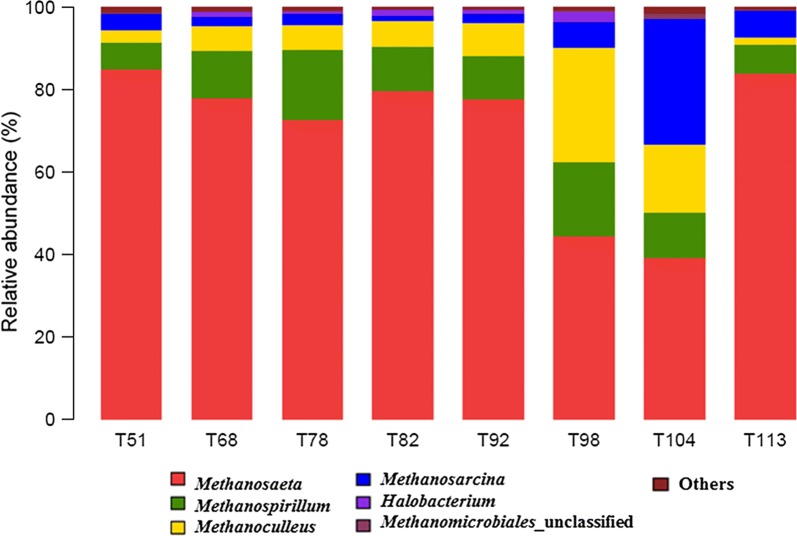
Taxonomic compositions of methanogens at the genus level in each sample retrieved from pyrosequencing. The sample was named as in Fig. 2

16S rDNA gene targeted high throughput pyrosequencing was used to reveal the archaeal community dynamics of the reactor samples. It was shown that most of the sequence reads were identified as methanogens (more than 98% in each individual sample). The results obtained from the application of diversity estimators of Chao 1, ACE and Shannon index are shown in Table 2 and the sequence distribution at the genus level is shown in Fig. 4. It was indicated that the diversity of bacterial community was much higher than archaeal community, which was caused by the inherent low abundance and diversity of the archaeal community.

It was observed that *Methanosaeta* was the most dominant genus, the relative concentration of which decreased from 82% on day 51 to 45% by 98st day. On the other hand, the relative concentration of *Methanosarcina* was found to increase from ~ 4 to 30% by day 104. These results indicate that the acetoclastic methanogens were the most dominant trophic type of methanogens present in the digester. *Methanosaeta*, the only known specialized acetoclastic methanogen (Garcia et al. 2000), are known to have lower maximum growth rate on acetate, but they are known to possess higher affinity for acetate as compared to the *Methanosarcina* (Conklin et al. 2006). It was thus suggested that higher acetate concentrations in the growth environment would favourable for the *Methanosarcina* growth, while lower concentrations would favourable for the *Methanosaeta* growth.

On 104th day, as the accumulated VFA increased to 700 mg L^−1^, the relative concentration of genus *Methanosarcina* was also found to increase to 30.45%. It was reported that *Methanosarcina* spp. grow in aggregates and form irregular cell clumps, which may result in increased tolerance against high concentrations of toxic agents (Calli et al. 2005). Furthermore, *Methanosarcina* are known to generate methane from acetate, methanol, monomethylamine, dimethylamine, trimethylamine, H_2_/CO_2_ and CO which are both acetoclastic and hydrogenotrophic (Conklin et al. 2006). Flexibility in metabolism and the special morphological characteristics of the members of *Methanosarcina* enable them to outcompete other methanogens during the acclimation phase. Other methanogens that were identified in the study were mostly assigned to genera *Methanospirillum* and *Methanoculleus*, indicating that the roles of hydrogenotrophic methanogens in FOG anaerobic digestion reactors are weaker than the acetoclastic methanogens.

### Linkage between the dynamics of microbial communities and reactor performances

**Fig. 5:**
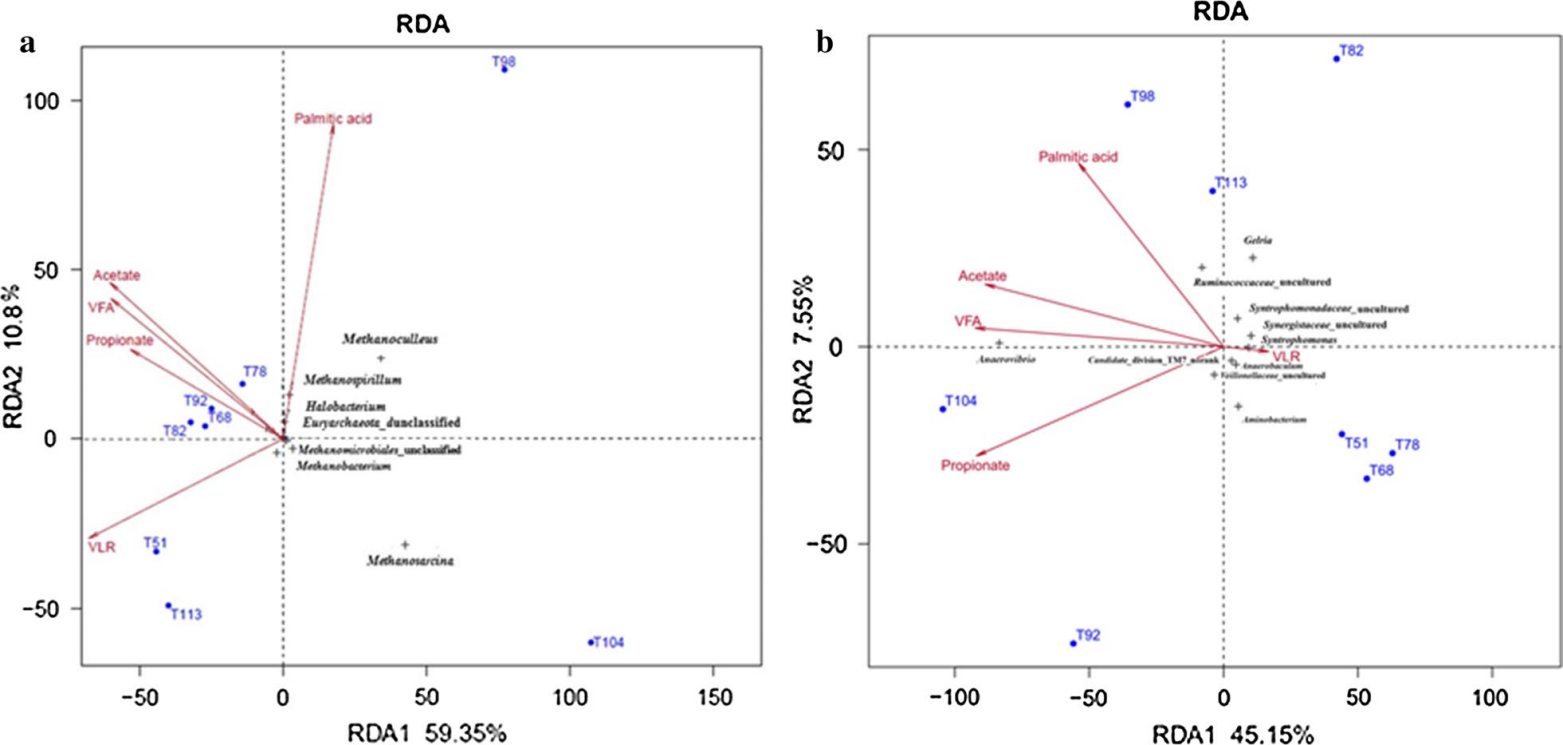
Triplots of RDA ordination diagrams of TOP10 archaeal community (**a**) and TOP10 bacterial community (**b**) with total VFA, acetate, propionate, palmitic acid and volumetric organic loading rate (here VLR = OLR)

Redundancy analysis (RDA) is a kind of sorting method that is based on correspondence analysis. It is mainly used to reflect the relationship between the bacteria and the environmental factors. The data presented in Fig. 5 shows that RDA was used to illustrate the relationships among bacterial community structures, reactor performances (including total VFA, acetate, propionate, palmitic acid) and operational conditions (volumetric organic loading). The fractions of the total variabilities that were illustrated using the RDA models were 45.15 and 59.35% for the bacteria and archaea OUT abundance datasets, respectively.

Redundancy analysis (Fig. 5) revealed that the TOP 10 archaeal communities in the initial stages of fermentation (T68, T78, T82, T92) were associated with total VFA, acetate, palmitic acid and volumetric organic loading rate (OLR). On the other hand, TOP 10 bacterial communities found in the later stages of fermentation (T98, T104, T113) were correlated with total VFA, acetate, propionate and palmitic acid. It was suggested that the effects of OLR were slightly on the archaeal communities (Fig. 5a), but it had profound effects on the bacterial communities (Fig. 5b). Palmitic acid contributed to the variability in bacterial community to some extent but not very significantly.

## Discussion

FOG is neither easily treated by conventional method, nor decomposed biologically (Stoll and Gupta 1997). FOG co-digestion with other waste materials was a feasible way to alleviate LCFA suppresses, but loading threshold values still was a barrier during FOG AD. FOG, as an AD material, is widely used for co-digestion with various other organic wastes (such as sludge and livestock manure). It has earlier been observed that high FOG loading rates cause technical discrepancies in the process of fermentation and even delay process recovery, especially due to the inhibitory effect of LCFAs. Hence, previous studies about the functional role and dynamics of the various microbial species that are involved in the LCFA *β*-oxidation in anaerobic reactors have been done (Table 3).

**Table 3:** Characterization of microbial community of AD with LCFA or FOG

References	Substrate	AD pattern	Method	Methanogenic archaea community	Bacterial community
Shigematsu et al. (2006)	Oleic and palmitic acids	Semi-continuous, CSTR, 37 °C	DGGE	Dominant genera *Methanosaeta* *Methanosarcina* * Methanospirillum*	Dominant phyla *Bacteroidetes* *Spirochaetes syntrophomonadaceae*
Sousa et al. (2007b)	MixLCFA, Palmitate 32–48%; Myristate 11–15%; oleate 23–26%	Batch, 35 °C	DGGE	Dominant genera *Methanosaeta* *Methanosarcina*	Dominant phyla(80%) *Clostridiaceae*
Baserba et al. (2012)	Oleate	Semi-continuous, CSTR, 55 °C	DGGE	Dominant genera *Methanosarcina* *Methanococcus*	Dominant phyla *Firmicutes* *Bacteroidetes* *Proteobacteria* *Thermotogae*
Yang et al. (2016)	FOG and sewage sludge	Semi-continuous, CSTR, 35 °C	High-throughput pyrosequencing	Dominant genera *Methanosarcinales* (11.7%) *Methanosaeta* (13.2%)	Dominant phyla: *Actinobacteria* (28.4%) *Firmicutes* (22.9%) *Bacteroidetes* (12.5%)
Ziels et al. (2016)	FOG and municipal sludge	Semi-continuous, CSTR, 35 °C	High-throughput pyrosequencing +qPCR	Dominant genera *Methanosaeta* (23 → 45%) *Methanospirillum* (1.3 → 34%) *Methanosphaera* (0 → 7%)	Dominant genera *Syntrophomonas* (1.2 → 9.0%) *Gelria*
Present study	FOG solely	Semi-continuous, CSTR, 35 °C	High-throughput pyrosequencing	Dominant genera *Methanosaeta* (82 → 45 → 82%) *Methanoculleus* (4 → 25 → 2%) *Methanospirillum* (7 → 18 → 8%) *Methanosarcina* (4 → 40%)	Dominant genera *Syntrophomonas* (12 → 35%) *Anaerovibrio* (10 → 40 → 20%)

As discussed previously, LCFA inhibition exists in the anaerobic reactors and is quite difficult to be resolved. However, co-digestion of FOG with other feedstock is considered an effective method to resolve this problem. The results of co-digesting FOG with municipal sludge indicated that the relative concentration of the syntrophic genus *Syntrophomonas* increased to ~ 15% of the total bacterial community in the reactor (Ziels et al. 2016).

In this study, FOG degradation-related bacteria such as the hydrolytic bacteria, syntrophic bacteria and methanogenic archaea were investigated. Furthermore, the correspondence analysis of environmental factors with microorganism community was also elucidated. The results of the present study indicated that with the elevation of the loading rates, the relative concentration of the members of genus *Anaerovibrio* (lipid hydrolysis bacteria) also increased from 9.3 to 40%, while the relative concentration of the members of genus *Syntrophomonas* increased to ~ 29%. The present study also indicated that *Methanosaeta* and *Methanosarcina* were the most dominant methanogenic genera and acetate fermentation was the main pathway for methane formation in anaerobic digestion reactors using waste cooking oil as the only carbon source. Additionally, whether OLR inrease had slight influence on the acetate fermentation pathway.

**Fig. 6:**
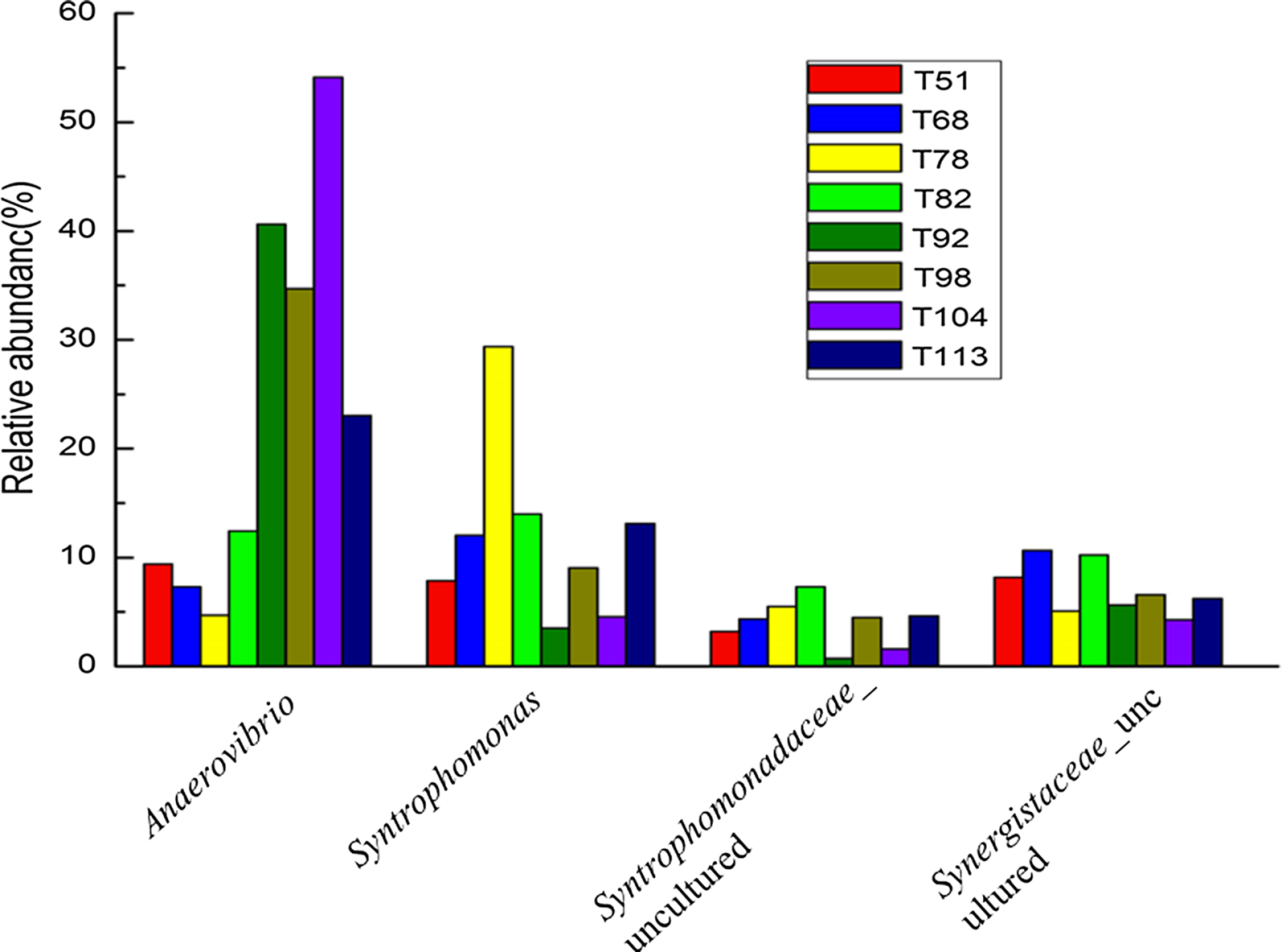
Relative abundance of the lipolytic bacteria and three mainly syntrophy bacteria at the time of 51st, 68th, 78th, 82nd, 92nd, 98th, 104th and 113th days

When the FOG loading rate was added to 2.0 g VS L^−1^ days^−1^, it came to the highest value for the proportion of *Anaerovibrio* (Fig. 6). *Anaerovibrio* is known as a fat decomposer that could hydrolyze triglycerides to produce glycerol and fatty acids. It could thus be concluded that the fat hydrolysis bacteria *Anaerovibrio* plays a key role in FOG anaerobic digestion.

*Syntrophomonas*, *Synergistaceae* and *Anaerovibrio* were the most dominant genera in the low organic loading phase (51st to 82nd day), and decreased when the organic loading rate increased (Fig. 6). The bacterial sequence library indicated that the concentration of *Syntrophomonas* increased from 7.8% in the initial phase to 29% by day 78 as the OLR increased. The concentration again went down to about 9% as the OLR reached 2.0 g VS L^−1^ days^−1^ (Fig. 6). The relative abundance of *Syntrophomonadaceae_*(uncultured) increased from ~ 3 to 7.3% by day 82, and then decreased to about 4.5% as the OLR reached 2.0 g VS L^−1^ days^−1^. *Syntrophomonadaceae* and *Syntrophaceae*, the members of syntrophic bacterial families, are well known for their LCFA *β*-oxidation properties (McInerney et al. 1981; Hatamoto et al. 2007; Sousa et al. 2007a). It can thus be implied that the members of these two syntrophic bacterial genera also play significant roles in anaerobic mesophilic digestion. However, the study indicated that their properties get compromised at high loading rates.

*Synergistaceae*_uncultured was found to remain at 6–10% as the loading rates increased. The members of *Synergistaceae* are known to belong to “Synergistes”. The species of this genus have been detected in samples which were collected from different environmental sources, such as anaerobic digesters, waste water, petroleum reservoirs, and soil (Sonia et al. 2007). The *Synergistaceae* was considered to play an intermediate role in the consortia, especially because they can use the amino acids made available from the breakdown of proteins by other organisms and provide short-chain fatty acids and sulphate for terminal degraders, such as methanogens and sulphate-reducing bacteria.

The present study suggested that a stable biogas production was obtained at an organic loading rate of 0.5–1.5 g VS L^−1^ days^−1^ using waste cooking oil skimmed from food waste as the only AD carbon source with a HTR of 10 days. Upon elevation of OLR, the genus *Methanosaeta* (acetoclastic methanogens) became the most dominant methanogen in the system. Syntrophic LCFA-degrading bacteria such as genera *Syntrophomona* and *Synergistaceae* were found to persist during the total AD process and hence resulted in better performance. The proportions of *Anaerovibrio* (lipids-degrading bacteria) were also found to increase up to 40% with OLR elevation. The effects of OLR on the bacterial community dynamics were found to be highly significant, as compared with that on the archaeal communities.

## Abbreviations

FW: food waste
LCFA: long chain fatty acids
AD: anaerobic digestion
FOG: fats, oils and grease
OLR: organic loading rate
SBY: specific biogas yield
SMY: specific methane yield
VFA: volatile fatty acids
TS: total solids
VS: volatile solid content
HRT: hydraulic retention time
RDA: redundancy analysis

## References

[CR1] Agency, U.S.E.P (2012). Municipal solid waste generation, recycling, and disposal in the United States: facts and figures for 2012.

[CR2] Angelidaki I, Ahring BK (1992). Effects of free long-chain fatty acids on thermophilic anaerobic digestion. Appl Microbiol Biotechnol.

[CR3] AWWA, APHA, WEF (1995). Standard methods for the examination of water and waste water.

[CR4] Baserba MG, Angelidaki I, Karakashev D (2012). Effect of continuous oleate addition on microbial communities involved in anaerobic digestion process. Bioresour Technol.

[CR5] Calli B, Mertoglu B, Inanc B, Yenigun O (2005). Community changes during startup in methanogenic bioreactors exposed to increasing levels of ammonia. Environ Technol.

[CR6] Christ O, Wilderer PA, Angerhöfer R, Faulstich M (2000). Mathematical modeling of the hydrolysis of anaerobic processes. Water Sci Technol.

[CR7] Cirne DG, Paloumet X, Bjornsson L, Alves MM, Mattiasson B (2007). Anaerobic digestion of lipid-rich wasted effects of lipid concentration. Renew Energy.

[CR8] Conklin A, Stensel HD, Ferguson J (2006). Growth kinetics and competition between *Methanosarcina* and *Methanosaeta* in mesophilic anaerobic digestion. Water Environ Res.

[CR9] Dasgupta BV, Mondal MK (2012). Bioenergy conversion of organic fraction of Varanasi municipal solid waste. Energy Proc.

[CR10] Davidsson A, Lovstedt C, Jansen JL, Gruvberger C, Aspegren H (2008). Codigestion of grease trap sludge and sewage sludge. Waste Manag.

[CR11] Finlay BJ, Maberly SC, Cooper JI (1997). Microbial diversity and ecosystem function. Oikos.

[CR12] Garcia JL, Patel BKC, Olivier B (2000). Taxonomic, phylogenetic, and ecological diversity of methanogenic archaea. Anaerobe.

[CR13] Girault R, Bridoux G, Nauleau F, Poullain C, Buffet J, Peu P, Sadowski AG, Beline F (2012). Anaerobic co-digestion of waste activated sludge and greasy sludge from flotation process: batch versus CSTR experiments to investigate optimal design. Bioresour Technol.

[CR14] Hanaki K, Matsuo T, Nagase M (1981). Mechanism of inhibition caused by longchain fatty acids in anaerobic digestion process. Biotechnol Bioeng.

[CR15] Hatamoto M, Imachi H, Fukayo S, Ohashi A, Harada H (2007). *Syntrophomonas palmitatica* sp. nov., an anaerobic, syntrophic, long-chain fatty-acid-oxidizing bacterium isolated from methanogenic sludge. Int J Syst Evol Microbiol.

[CR16] Jackson BE, Bhupathiraju VK, Tanner RS, Woese CR, McInerney MJ (1999). *Syntrophus aciditrophicus* sp. nov., a new anaerobic bacterium that degrades fatty acids and benzoate in syntrophic association with hydrogen-using microorganisms. Arch Microbiol.

[CR17] Kim JK, Han GH, Oh BR, Chun YN, Eom C, Kim SW (2008). Volumetric scale-up of a three stage fermentation system for food waste treatment. Bioresour Technol.

[CR18] Lalman JA, Bagley DM (2000). Anaerobic degradation and inhibitory effects of linoleic acid. Water Res.

[CR19] Lin CSK, Pfaltzgraff LA, Herrero-Davila L, Mubofu EB, Abderrahim S, Clark JH, Koutinas AA, Kopsahelis N, Stamatelatou K, Dickson F, Thankappan S, Mohamed Z, Brocklesby R, Luque R (2013). Food waste as a valuable resource for the production of chemicals, materials and fuels. Current situation and global perspective. Energy. Environ Sci.

[CR20] Luostarinen S, Luste S, Sillanp M (2009). Increased biogas production at wastewater treatment plants through co-digestion of sewage sludge with grease trap sludge from a meat processing plant. Bioresour Technol.

[CR21] McInerney MJ (1992). The genus *Syntrophomonas*, and other syntrophic bacteria. Prokaryotes.

[CR22] McInerney MJ, Bryant MP, Hespell RB, Costerton JW (1981). *Syntrophomonas wolfei* gen. nov. sp. nov., an anaerobic, syntrophic, fatty acid-oxidizing bacterium. Appl Environ Microbiol.

[CR23] Meng Y, Shen F, Yuan H, Zou D, Liu Y, Zhu B, Chufo A, Jaffar M, Li X (2014). Start-up and operation strategies on the liquefied food waste anaerobic digestion and a full-scale case application. Bioprocess Biosyst Eng.

[CR24] Noutsopoulos C, Mamais D, Antoniou K, Avramides C, Oikonomopoulos P, Fountoulakis I (2013). Anaerobic co-digestion of grease sludge and sewage sludge: the effect of organic loading and grease sludge content. Bioresour Technol.

[CR25] Pereira MA, Pires OC, Mota M, Alves MM (2005). Anaerobic biodegradation of oleic and palmitic acids: evidence of mass transfer limitations caused by long chain fatty acid accumulation onto the anaerobic sludge. Biotechnol Bioeng.

[CR26] Rinzema A, Boone M, Knippenberg K, van Lettinga G (1994). Bactericidal effect of long chain fatty acids in anaerobic digestion. Water Environ Res.

[CR27] Shigematsu T, Tang Y, Mizuno Y, Kawaguchi H, Morimura S, Kida K (2006). Microbial diversity of mesophilic methanogenic consortium that can degrade long-chain fatty acids in chemostat cultivation. J Biosci Bioeng.

[CR28] Silva SA, Cavaleiro AJ, Pereira MA, Stams AJM, Alves MM, Sousa DZ (2014). Long-term acclimation of anaerobic sludges for high-rate methanogenesis from LCFA. Biomass Bioenergy.

[CR29] Silvestre G, Rodríguez-Abalde A, Fern_andez B, Flotats X, Bonmatí A (2011). Biomass adaptation over anaerobic co-digestion of sewage sludge and trapped grease waste. Bioresour Technol.

[CR30] Sonia RV, Richard MP, William GW (2007). The division “*Synergistes*”. Anaerobe.

[CR31] Sousa DZ, Smidt H, Alves MM, Stams AJM (2007). *Syntrophomonas zehnderi* sp. nov., an anaerobe that degrades long-chain fatty acids in co-culture with *Methanobacterium formicicum*. Int J Syst Evol Microbiol.

[CR32] Sousa DZ, Pereira MA, Smidt H, Stams AJM, Alves MM (2007). Molecular assessment of complex microbial communities degrading long chain fatty acids in methanogenic bioreactors. FEMS Microbiol Ecol.

[CR33] Stoll U, Gupta H (1997). Management strategies for oil and grease residues. Waste Manag Res.

[CR34] Sun Y, Wang D, Yan J, Qiao W, Wang W, Zhu T (2014). Effects of lipid concentration on anaerobic co-digestion of municipal biomass wastes. Waste Manage.

[CR35] Wan C, Zhou Q, Fu G, Li Y (2011). Semi-continuous anaerobic co-digestion of thickened waste activated sludge and fat, oil and grease. Waste Manag.

[CR36] Wang L, Aziz TN, de los Reyes FL (2013). Determining the limits of anaerobic codigestion of thickened waste activated sludge with grease interceptor waste. Water Res.

[CR37] Wu C, Dong X, Liu X (2007). *Syntrophomonas wolfei* subsp. *Methylbutyratica* subsp. nov., and assignment of *Syntrophomonas wolfei* subsp. saponavida to *Syntrophomonas saponavida* sp. nov. comb. nov. Syst Appl Microbiol.

[CR38] Yang ZH, Xu R, Zheng Y, Chen T, Zhao LJ, Li M (2016). Characterization of extracellular polymeric substances and microbial diversity in anaerobic co-digestion reactor treated sewage sludge with fat, oil, grease. Bioresour Technol.

[CR39] Zhang C, Xiao G, Peng L, Su H, Tan T (2013). The anaerobic co-digestion of food waste and cattle manure. Bioresour Technol.

[CR40] Ziels RM, Karlsson A, Beck DAC, Ejlertsson J, Yekta SS, Bjorn A, Stensel HD, Svensson BH (2016). Microbial community adaptation influences long-chain fatty acid conversion during anaerobic codigestion of fats, oils, and grease with municipal sludge. Water Res.

